# Multiple micronutrient deficiencies alter energy metabolism in host and gut microbiome in an early-life murine model

**DOI:** 10.3389/fnut.2023.1151670

**Published:** 2023-07-10

**Authors:** Paula T. Littlejohn, Haggai Bar-Yoseph, Karlie Edwards, Hong Li, Cynthia Y. Ramirez-Contreras, Ravi Holani, Avril Metcalfe-Roach, Yiyun M. Fan, Tom Min-Shih Yang, Nina Radisavljevic, Xiaoke Hu, James D. Johnson, B. Brett Finlay

**Affiliations:** ^1^Michael Smith Laboratories, University of British Columbia, Vancouver, BC, Canada; ^2^Department of Microbiology and Immunology, University of British Columbia, Vancouver, BC, Canada; ^3^Department of Medical Genetics, Faculty of Medicine, University of British Columbia, Vancouver, BC, Canada; ^4^Life Sciences Institute and Department of Cellular and Physiological Sciences, University of British Columbia, Vancouver, BC, Canada; ^5^Department of Pediatrics, University of British Columbia, Vancouver, BC, Canada; ^6^Department of Cellular and Physiological Sciences, University of British Columbia, Vancouver, BC, Canada; ^7^Department of Biochemistry and Molecular Biology, University of British Columbia, Vancouver, BC, Canada

**Keywords:** multiple micronutrient deficiencies, glucose dysregulation, stunting, gut microbiome, insulin dysregulation

## Abstract

**Introduction:**

Micronutrients perform a wide range of physiological functions essential for growth and development. However, most people still need to meet the estimated average requirement worldwide. Globally, 2 billion people suffer from micronutrient deficiency, most of which are co-occurring deficiencies in children under age five. Despite decades of research, animal models studying multiple micronutrient deficiencies within the early-life period are lacking, which hinders our complete understanding of the long-term health implications and may contribute to the inefficacy of some nutritional interventions. Evidence supporting the Developmental Origins of Health and Disease (DOHaD) theory demonstrates that early-life nutritional deficiencies carry life-long consequences mediated through various mechanisms such as abnormal metabolic programming, stunting, altered body composition, and the gut microbiome. However, this is largely unexplored in the multiple micronutrient deficient host.

**Methods:**

we developed a preclinical model to examine undernutrition’s metabolic and functional impact on the host and gut microbiome early in life. Three-week-old weanling C57BL/6N male mice were fed a low-micronutrient diet deficient in zinc, folate, iron, vitamin A, and vitamin B12 or a control diet for 4-weeks.

**Results:**

Our results showed that early-life multiple micronutrient deficiencies induced stunting, altered body composition, impaired glucose and insulin tolerance, and altered the levels of other micronutrients not depleted in the diet within the host. In addition, functional metagenomics profiling and a carbohydrate fermentation assay showed an increased microbial preference for simple sugars rather than complex ones, suggestive of a less developed microbiome in the low-micronutrient-fed mice. Moreover, we found that a zinc-only deficient diet was not sufficient to induce these phenotypes, further supporting the importance of studying co-occurring deficiencies.

**Discussion:**

Together, these findings highlight a previously unappreciated role of early-life multiple micronutrient deficiencies in shaping the metabolic phenome of the host and gut microbiome through altered glucose energy metabolism, which may have implications for metabolic disease later in life in micronutrient-deficient survivors.

## Introduction

Approximately thirty micronutrients (i.e., vitamins and minerals) are considered essential for general health and normal physiological function at varying levels over the life course (1). Unfortunately, much of the global population fails to meet the estimated average requirement (2–4). More than 2 billion people worldwide suffer from one or more micronutrient deficiencies, the main nutrients being iron, iodine, zinc, vitamins A, and B. Current estimates show that micronutrient deficiencies affect more than half of all children (372 million) under the age of five across the globe, with most experiencing deficiencies in more than one critical micronutrient (5). This number has risen and will continue to rise due to food disruptions caused by the COVID-19 pandemic and the Ukrainian war. Decades of micronutrient deficiency studies have focused primarily on single nutrients (2). Yet, “A characteristic feature of nutritional deficiencies in humans is their multiple nature. Single deficiencies are uncommon, multiple deficiencies are the rule” (6). Concurrent micronutrient deficiencies have been linked to impaired growth, morbidity, and mortality in children living in low-to-mid income countries (LMICs) (7, 8). A survey of a cohort of Indonesian pregnant women and their offspring found a high prevalence of concurrent deficiencies, including iron, zinc, and vitamin A (9). Most of the infants were anemic and stunted. Similarly, in a study of 243 infection-free Mongolian children aged 6–36 months from 8 different regional locations, 78% presented with two or more concurrent micronutrient deficiencies (10). When measured in the liver, significant concurrent deficiencies were found in selenium, iron, folate, zinc, retinol (i.e., vitamin A), and vitamin D.

Vulnerability to micronutrient deficiencies remains throughout life. However, nutritional deficiencies that occur during infancy and childhood confer immediate and life-long consequences (11–17). This early-life window is marked by a period of intense growth, development, and metabolic programming of various organ systems, including the brain and bone, which requires substantial nutritional input (11–17). Considerable evidence shows that early-life nutritional insults increase disease risk later in life through various mechanisms, including gut microbiome alteration, epigenetic modulation, and metabolic programming (14, 16). Nevertheless, concurrent micronutrient deficiencies remain a grossly neglected area of research. Data collection is suboptimal, and micronutrient supplements show only marginal success (18). Studies have shown that protein malnutrition survivors (e.g., kwashiorkor and marasmus) have an increased risk of metabolic disease later in life through altered energy metabolism. However, this is understudied in micronutrient-deficient survivors, and the mechanisms involved are not fully understood (19, 20).

In addition, substantial evidence shows that the first 1,000 days of life coincide with the assembly and maturation of the intestinal microbiome, making it sensitive to nutritional programming (16). Symbiotically co-existing with its host, the microbiome extends our metabolic capacity by facilitating digestion of insoluble fibers, nutrient absorption, degradation of drugs, and biosynthesis of certain vitamins and amino acids (16, 21, 22). Recently, the microbial contribution to malnutrition and stunting has been recognized by describing the malnourished microbiome. However, these studies have primarily centered around protein malnutrition (23). Studies focusing on single micronutrients have resulted in limited data showing that bidirectional relationships between gut microbiota populations and micronutrients can occur (24, 25). Hibberd and colleagues investigated the role of single and multiple micronutrients (i.e., vitamin A, folate, iron, and zinc) in modulating the composition, meta-transcriptome, and molecular function of the gut microbiota in mice using a human bacterial consortium of 92 species in adult gnotobiotic mice (26). The authors found that both vitamin A and the multiple micronutrient-deficient diet significantly altered the relative abundance of *B. vulgatus* and *B. dorei* (26). No difference in body weight was found. However, this model focused on adult mice and not the early life window. Furthermore, the functional metabolic maturation of the microbiome that happens in tandem with community assembly has received little attention. In infancy, early colonization of *Enterobacteriaceae* is associated with the metabolic repertoire to degrade simple sugars, synthesize vitamins, engage in amino acid transport, and in creating a more aerobic environment to allow for colonization of other aerotolerant bacteria (21). As the microbiome reaches maturity, bacterial species with the capacity to degrade complex carbohydrates, namely fiber, to produce short-chain fatty acids, *de novo* biosynthesis of amino acids, and methane production begin to appear (21). This functional maturation, however, needs to be better studied in the undernourished host. Thus, animal models of early life cooccurring multiple micronutrient deficiencies are needed to shed additional light and advance our understanding of the life course impact of this deficiency on host and gut microbiome. Here, we report a new *in-vivo* model that addresses this knowledge gap and further investigates the hidden cost of early-life exposure to multiple micronutrient deficiencies. We chose to focus our study on vitamin A, vitamin B12, folate, zinc, and iron, as these have been identified as persistent global concerns by the World Health Organization and the Micronutrient Forum. We examined the impact of their deficiency on the host phenome (sum of phenotypes) and the functional metabolic capacity of the gut microbiome to better understand future disease risk. This model can be used experimentally to evaluate the interacting effects of multiple micronutrient deficiencies and help determine how this early life exposure shapes future health outcomes.

## Materials and methods

### Animals and model approach

All experiments were approved and performed in accordance with the University of British Columbia’s (UBC) Animal Care Committee (ACC) and Canadian Council on Animal Care (CCAC) ethics guidelines for research, training, and housing (Protocol A18-0227).

Weanling 3-week-old C57BL/6N male pups were purchased from Charles Rivers (Kingston, NY, USA). Mice were distributed equally by weight (*n* = 6–10) and housed 3–5 per cage in a 12-h light/dark cycle barrier animal vivarium. One group of mice (*n* = 6–10) received an experimental control diet (CON) and the other a multiple low-micronutrient (LM) treatment diet—deficient in vitamins A, vitamin B12, folate, zinc, and iron. Diets were isocaloric, based upon the standard AIN-93 formulation, comprising similar macronutrients (protein 20%, carbohydrates 65%, fat 15%) (D18062501I and D19041709I, respectively) (Research Diets Inc., New Brunswick, NJ, USA) (Table 1). Pectin was added to the LM diet to experimentally prevent coprophagic reabsorption of vitamin B12; however, the amount provided the same amount of fiber as cellulose in the CON diet. Diets were irradiated prior to shipment and stored at -20°C until use. Diets were brought to room temperature prior to use, and animals were given *ad libitum* access to their respective diets and water throughout the entire experiment. Mice remained on their respective diets for 28 days ± 2 (i.e., 4 weeks). Chow was weighed weekly for each group by cage using a standard scale (g), and total consumption per group was recorded.

**Table 1:** Dietary composition for the multiple low micronutrient and experimental control.

Ingredients	D18062501		D19041709	
	Control Diet		LM w/Pectin	
	gm%	kcal%	gm%	kcal%
Protein (egg white)	18.9	20	19.2	20
Carbohydrate	63.1	65	61.7	62
Fat	6.5	15	6.6	15
Total		100		100
kcal/gm	3.77		3.77	
Ingredient	gm	kcal	gm	kcal
Casein	0	0	0	0
Egg White	203	812	203	812
L-Cystine	0	0	0	0
Corn Starch	346	1,384	332.7	1330.8
Maltodextrin 10	45	180	45	180
Dextrose	250	1,000	250	1,000
Sucrose	0	0	0	0
Cellulose, BW200	75	0	22	0
Inulin	25	25	25	25
Pectin, Tic Gums	0	0	53	53
Soybean Oil	70	630	70	630
Mineral Mix S10026	10	0	0	0
Mineral Mix S19427 (No Ca, P, K, Zn, or Fe)	0	0	10	0
Dicalcium Phosphate	13	0	13	0
Calcium Carbonate	5.5	0	5.5	0
Potassium Citrate, 1 H2O	16.5	0	16.5	0
Ferric Citrate (17.4% Fe)	0	0	0.029	0
Zinc Carbonate (52.1% Zn)	0	0	0.004	0
Vitamin Mix V10001	10	40	0	0
Vitamin Mix V15927 (No vitamin A, Folate, or B12)	0	0	10	40
Vitamin Mix V15928 (350 IU A, 3 ug B12, 0.11 mg Folate)	0	0	0	0
Biotin, 1%	0.4	0	0.4	0
Choline Bitartrate	2	0	2	0
Pure Red Dye #40	0	0	0	0
Pure Blue Dye #1	0	0	0.05	0
Pure Yellow Dye #5	0.05	0	0	0
Total	1071.45	4071	1058.183	4071

Diet based on the AIN-93 formulation and modified for this experiment.

### Stunting and body composition

Stunting was measured using standard anthropometric measures. Bodyweight (g) was obtained at baseline (Day 0) and the end of 4-weeks (Day 28) using a calibrated scale. Tail length (cm) was measured after euthanasia using a standard ruler. Post euthanasia, the left skinless leg was collected and placed in a 50 mL falcon tube with 2% potassium hydroxide and shaken at 250–300 rpm for 1–4 days at 37°C. After all non-bone tissue was dissolved in the mixture, tibia length (mm) was measured using an electronic digital caliper (Neiko 01407A, Ridgerock Tools Inc., Gardena, CA, USA). Body weight (mg) to tibia length (mm) ratio was calculated to normalize growth.

A dual-energy X-ray absorptiometry (DEXA) scan to evaluate body composition was performed at baseline (Day 0) and endpoint (Day 28) using the PIXImus Mouse Densitometer (Lunar Corporation, Madison, WI, USA). A phantom mouse was used to calibrate the equipment as per the manufacturer’s instructions prior to starting the assessment. All mice were weighed prior to the start of the measurement. Mice were anesthetized using 2–4% isoflurane in oxygen with constant flow provided by nose cone throughout the measurement. Fully unconscious mice were placed in a prone position on the DEXA specimen tray, spine was fully extended in a straight line, legs were slightly outstretched, and heads were forward. Body weight, sex, and mouse identification were entered for each mouse prior to measurement. The scan was then performed and checked for accuracy. Region of interest (ROI) included full body. Image files were then processed to exclude the head region from analysis within the PIXImus software as described by the manufacturer. Reprocessed report files for each mouse were then printed for statistical analysis. Mice were then moved to a recovery cage free of any bedding on a heating pad and monitored until movements were normal prior to returning to their original cage. Raw values were corrected using the Mouse Metabolic Research Unit DEXA PIXImus correction calculator (27).

### Micronutrient assessment

Serum vitamin B12 and red blood cells folate (B9) Levels were assayed using by the Analytical Core for Metabolomics and Nutrition (ACMaN, Vancouver, BC) using the ARCHITECT i1000SR immunoassay analyzer (Abbott Laboratories, Abbott Park, IL, USA). Exactly 100 μL of whole blood was placed into a light-sensitive 1.5 mL tube (#Z688312, Millipore Sigma, Oakville, CA, USA) labeled containing 1,000 μL of 1% (w/v filtered distilled water) ascorbic acid buffer for folate analysis. Samples were incubated for 60 min at 37°C before freezing at −80°C to prevent degradation. Samples were automatically pre-treated by the ARCHITECT using the folate assay (Abbott, Wiesbaden, Germany), causing the liberation of folate from the folate-binding protein and subsequently replacing it with its own folate-binding protein coated with paramagnetic microparticles. Samples are washed and then treated with a pteroic acid-acridinium labeled conjugate, which binds to free binding sites on the microparticles. The bound conjugate was measured by chemiluminescence in relative light units yielding the total folate concentration. Similarly, for cobalamin analysis, samples were pre-treated by the ARCHITECT using the B12 Abbott assay (Wiesbaden, Germany), which was then combined with intrinsic factor coated microparticles. This binds all the B12 to any unoccupied binding sites on the microparticle. Samples were washed and then treated with B12 acridinium labeled conjugate. Chemiluminescence was measured to calculate B12 concentrations. According to the manufacturer’s instructions, serum ferritin was measured using the Mouse Ferritin ELISA kit (#MBS564067, MyBioSource Inc., San Diego, CA, USA). Vitamin A esters (retinol/retinal) in the liver were measured using HPLC mass spectrometry. Liver samples were collected in 1.5 mL LightSafe microcentrifuge tubes to prevent the leaching of retinoids (#Z688312, Millipore Sigma, Oakville, CA, USA). Hepatic storage concentrations of zinc, iron, and all other metals (i.e., copper, selenium) were measured by the Analytical Chemistry Laboratory (British Columbia Institute of Technology, Burnaby, BC, Canada) using an Agilent 8,900 (Agilent Technologies, Santa Clara, CA, USA) inductively coupled plasma triple quadrupole mass spectrometry (ICP-MS MS). Liver samples were collected using a 50 mL metal-free tube (DigiTUBE #010–500-263, Quebec City, Canada) to prevent metal contamination.

### Complete blood count panel

Approximately 300 μL of whole blood was collected from mice in an ethylenediaminetetraacetic acid (EDTA) precoated tube and allowed to sit at room temperature for 1 h, then transferred to 4°C until sample drop off. All samples were processed at the Diagnostic Laboratory at the University of British Columbia Centre for Comparative Medicine Animal Care Services.

### Hormone and enzyme assays

Serum insulin level obtained was determined using blood collected from the saphenous vein in 2 mL heparin-coated Eppendorf tubes and measured using an ultrasensitive mouse insulin ELISA Kit in a semi-fasted state (i.e., removal of food from the cage) (#80-INSMSU-E01/E10, ALPCO, Salem, New Hampshire, USA). Glycogen was measured using frozen liver samples according to the manufacturer’s instructions (Cayman Chemicals, Ann Arbor, MI, USA). Glucagon (Crystal Chemicals, Elk Grove Village, IL, USA) concentrations were determined using commercially available kits (Pro-insulin: #10–1,232-01, Mercodia, Winston Salem, NC, insulin-degrading enzyme (IDE): LS-F5702, LSBio, Seattle, WA, USA, and c-peptide:#80-CPTMS-E01, ALPCO, Salem, New Hampshire, USA). Serum samples were diluted per kit recommendation, if applicable. All samples were read at OD 450 nm using BioTek microplate reader (Santa Clara, CA, USA). Total serum concentration of Insulin-like growth factor-1 (IGF-1) was measured using Mouse IGF-1 ELISA Kit (#RAB (1)0229, MilliporeSigma, Oakville, ON, Canada). Samples were prepared following manufacturers’ standard procedures and read at OD 450 nm on the BioTek microplate reader (Santa Clara, CA, USA).

### Glucose and insulin tolerance tests

At the end of the experiment (Day 28), mice were fasted for 4 h (i.e., food removed and cage changed). Fasting glucose was measured from blood collected from mouse tail vein using the Accu-Chek Guide glucometer (Roche Diabetes Care, Indianapolis, IN, USA). Additionally, glucose metabolism was assessed by intraperitoneal glucose tolerance test (IPGTT). After a 4 h fast, baseline (i.e., fasting) glucose was obtained (time 0). Mice received an IP injection of D-dextrose (fresh sterile D-(+)-glucose solution 2 g/kg body weight [#D9434, MilliporeSigma, Oakville, ON, Canada] dissolved in 0.9% NaCl, physiological saline [#S9888, MilliporeSigma, Oakville, ON, Canada]). Blood glucose concentrations were measured at times 15-, 30-, 60-, 90-, and 120-min post IP injection using a glucometer (Accu-Chek Guide glucometer, Roche Diabetes Care, Indianapolis, IN, USA).

One week later, the effectiveness of glucose clearance in response to exogenous insulin *via* intraperitoneal insulin tolerance test (IPITT) was performed. Briefly, mice were fasted as before and intraperitoneally injected with insulin 0.75 U/kg body weight in 1X PBS (Humalog insulin lispro for injection, Eli Lilly, Toronto, ON, Canada). Blood glucose concentration was determined at baseline (time 0), then 15-, 30-, 60-, 90-, and 120-min post-injection using Accu-Chek Guide glucometer. Repeated samples were taken at the same time point for both tests over four independent experiments.

### Targeted metabolomics

We used targeted metabolomics to assess the impact of the diet on lipid profile in mice. Day 28 serum samples were aliquoted and sent to the Analytical Core for Metabolomics and Nutrition (ACMaN, Vancouver, BC) for processing. A total of thirty-one non-esterified free fatty acids (NEFA) were analyzed, and significant results presented. Esterified and non-esterified free fatty acids (FFAs) were assessed in serum using gas chromatography with a flame ionization detector (GC-FID). Total lipids were transmethylated in methanol with boron trifluoride (14%) at 100C × 20 min. After cooling, 3 mL of 0.9% NaCl and 6 mL hexane were added to water and vortexed. The upper hexane layer was removed, transferred to a clean tube, and remaining solvent evaporated under nitrogen. Dried sample residue was reconstituted in hexane and injected into the GC-FID using Supelco SP2380 30 m × 0.25 mm inner diameter and 250 μm thick-film columns for quantification (Millipore-Sigma). Individual fatty acids were identified by comparison to authentic standards purchased from Millipore Sigma and Nu-chek Prep (Minnesota, USA). Peak areas for each fatty acid were used to calculate the percent weight of total fatty acids.

### Microbiome functional analysis

Fecal pellets were collected under clean conditions on Day 0 and Day 28 and immediately placed in an Eppendorf tube on dry ice, then transferred to a −80°C freezer until sequencing. DNA extraction and metagenomic sequencing was performed by Microbiome Insights (Vancouver, BC) as described previously (manuscript under revision). We used whole genome shotgun sequencing to characterize the gut microbial metagenome at Days 0 and 28 (manuscript under revision elsewhere). High-quality reads from both time points were used for hierarchal functional annotation using Super-Focus, Kraken, and SEED databases, allowing us to examine functional changes in response to the dietary treatment pre and post-treatment. Data were normalized to percent abundance. Reads that mapped to sugar utilization were summed by category (mono-, di-, polysaccharide) and by individual sugars. Gene abundances at Day 0 and at Day 28 were compared between CON and LM groups using Wilcoxon rank-sum tests. *p* values of individual sugars were grouped by category and corrected using the Benjamini-Hochberg (FDR) method. Data were analyzed using R (version 4.1.0).

### Microbiome carbohydrate fermentation assay

Bacterial glycerol stock from Day 28 fecal samples was first cultured in fastidious anaerobic broth overnight at 37°C under anaerobic conditions. Bacteria concentration was determined by OD_600_ and inoculum diluted to 10^9^ cells/mL. Carbohydrate media was made by preparing 4.5 mL of Phenol Red Broth Base (Phenol red as pH indicator) in a standard test tube. Durham tubes were inversely placed within the test tube (upside down) into the broth, taking care to cover the entire Durham tube with the broth. Media tubes were then autoclaved at 121°C, 16 psi for 20 min to remove any air. Sugar broth media was inoculated with 10^9^ cells/mL fecal inoculum and incubated overnight at 37°C nonshaking. We used a standard ruler to measure the size of the gas bubbles in millimeters.

### Short-chain fatty acids and correlation analysis

Short-chain fatty acid (SCFA) analysis was done by Microbiome Insights (Vancouver, BC) and followed methods similar to Zhao et al. (28) Briefly, fecal pellets from Day 28 were resuspended in MilliQ-grade H2O, and homogenized using MP Bio FastPrep, for 1 min at 4.0 m/s. 5 M HCl was added to acidify fecal suspensions to a final pH of 2.0. Acidified fecal suspensions were incubated for five minutes and centrifuged at 10,000 RPM to separate the supernatant. Fecal supernatants were spiked with 2-Ethylbutyric acid for a final concentration of 1 mM. Extracted SCFA supernatants were stored in 2-ml GC vials with glass inserts. SCFA were detected using gas chromatography (Thermo Trace 1,310), coupled to a flame ionization detector using Thermo TG-WAXMS A GC Column, 30 m, 0.32 mm, 0.25 um’ columns (Thermo). Detection settings for the flame ionization detector were set to 240°C, hydrogen 35.0 mL/min, air 350.0 mL/min, and makeup gas (nitrogen) 40.0 mL/min. The oven temperature gradient was 100 to 180°C with a total run of 10 min. SCFA standards (Sigma-Aldrich, Oakville, ON, Canada) acetic acid (LabChem LC101001, CAS #64-19-7), propionic acid (#94425-5ML-F), isobutyric acid (#11754-100ML), butyric acid (#B103500-100ML), isovaleric acid (#129542-100ML), valeric acid (#240370-5ML), hexanoic acid (#153745-100G), heptanoic acid (#75190-100ML) and 2-ethylbutyric acid (#109959-100ML) were injected at every run. Concentrations were normalized to the amount of input material (mmol SCFA/ kg mouse feces). Valeric acid, hexanoic acid, heptanoic acid, and 2-ethylbutyric acid levels were under the detection limit and were excluded from further analysis. The correlation of diet with the concentration of each SCFA was determined using Wilcoxon rank-sum tests, and *p* values were corrected via the Benjamini-Hochberg method where appropriate and represented as q values. Hexanoate and valerate were undetectable. Differences in SCFA concentrations (mM/L) between CON and LM groups were calculated for each SCFA using Wilcoxon rank-sum tests, and *p* values were FDR corrected. For each dietary group, SCFAs with a corrected value of *p* of <0.10 were correlated with fasting glucose using Spearman’s rank correlation. *p* values for all tested SCFAs were corrected separately for each dietary group.

### Zinc deficiency experiment

Three-week-old weanling male C57BL/6N mice were purchased from Charles Rivers and were fed either a CON, LM, or low-zinc (ZND) diet for 4 weeks, as described above. The zinc diet was isocaloric to the CON and LM and contained protein 20%, carbohydrates 65%, fat 15%, and standard micronutrients with only 2 mg of zinc (D21051302I, Research Diets Inc., New Brunswick, NJ, USA). Diet was irradiated prior to shipment and stored at -20°C until use (Supplementary Table S3).

### Statistical analyses

Statistical significance testing between the two dietary groups (CON vs. LM) was done using a two-tailed Student’s t-test. Two-way ANOVA with Šidák multiple comparison testing was used for body composition analysis. Glucose and insulin data were analyzed using area under the curve (AUC) and one-way ANOVA with *post hoc* Tukey’s test. Food consumption between groups was compared using Mann–Whitney test. Analysis was done using GraphPad Prism software version 9 (San Diego, CA, USA). *p* value of <0.05 were considered statistically significant. Statistically significant *p* values were reported as *p < 0.05, **p < 0.01, ***p < 0.001, ****p < 0.0001, ns (not significant) *p* value >0.05. Data is represented as ±SEM unless otherwise indicated.

## Results

### Murine model of multiple micronutrient deficiencies results in anemia

**Figure 1:**
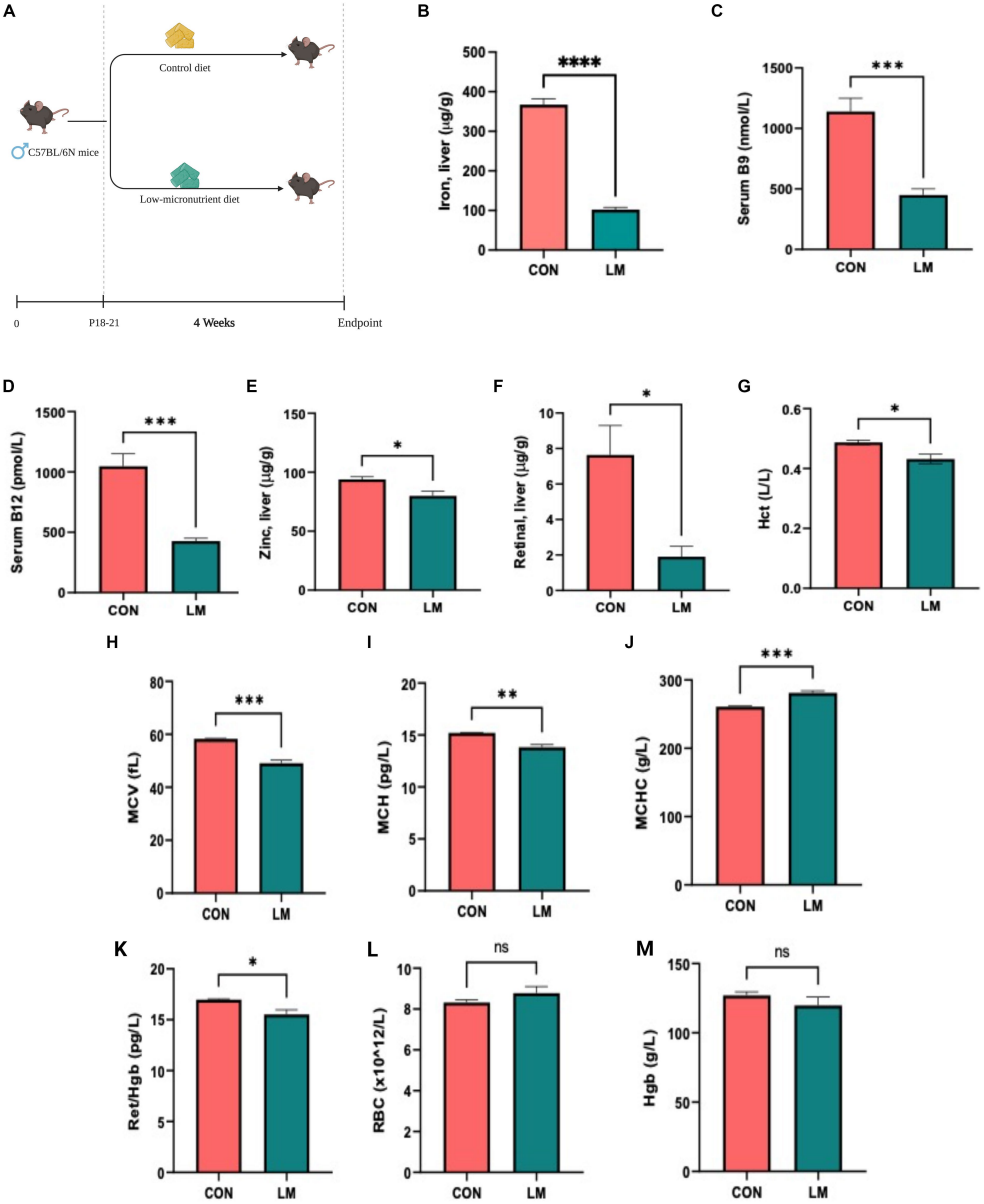
Model design, micronutrient depletion and impact on host physiology. **(A)** Newly weaned 3-week-old C57BL/6N mice were placed on a multiple low-micronutrient (LM) or experimental control (CON) diet for four weeks. **(B–F)** Micronutrients [iron in liver tissues, serum folate and vitamin B12, retinal (vitamin A)] and zinc in liver were markedly lower in LM mice. **(G–M)** Complete blood count: hematocrit (Hct), mean corpuscular volume (MCV), mean corpuscular hemoglobin (MCH), mean corpuscular hemoglobin concentration (MCHC), reticulocyte to hemoglobin (Ret/hgb) ratio revealed diet-induced anemia in the LM mice. Data analyzed using Student’s *t*-test and represented as mean ± SEM, *p* values expressed as *p < 0.05, **p < 0.01, ***p < 0.001, ****p < 0.0001, ns, not significant *p* > 0.05.

We first determined whether the diet successfully induced micronutrient deficiency in the treated mice. Our results showed stark depletion of all five micronutrients in the LM mice compared to controls (Figures 1A–F). Mice on the low-micronutrient diet presented with anemia, indicated by several markers on the complete blood count panel. Reticulocyte to hemoglobin ratio, a strong indicator of bone marrow malfunction in reticulocyte production and aplastic anemia, was also reduced in the low-micronutrient group. No difference was seen in red blood cell count and hemoglobin between the groups (Figures 1G–M). Intriguingly, we also found copper, selenium, and molybdenum depletion and elevated manganese, although these nutrients were unaltered in our dietary formulation (Supplementary Figure S1) in the LM group. Taken together, we successfully created a co-occurring multiple micronutrient deficient mouse model with induced anemia.

Impact of multiple micronutrient deficiencies on growth and body composition

**Figure 2:**
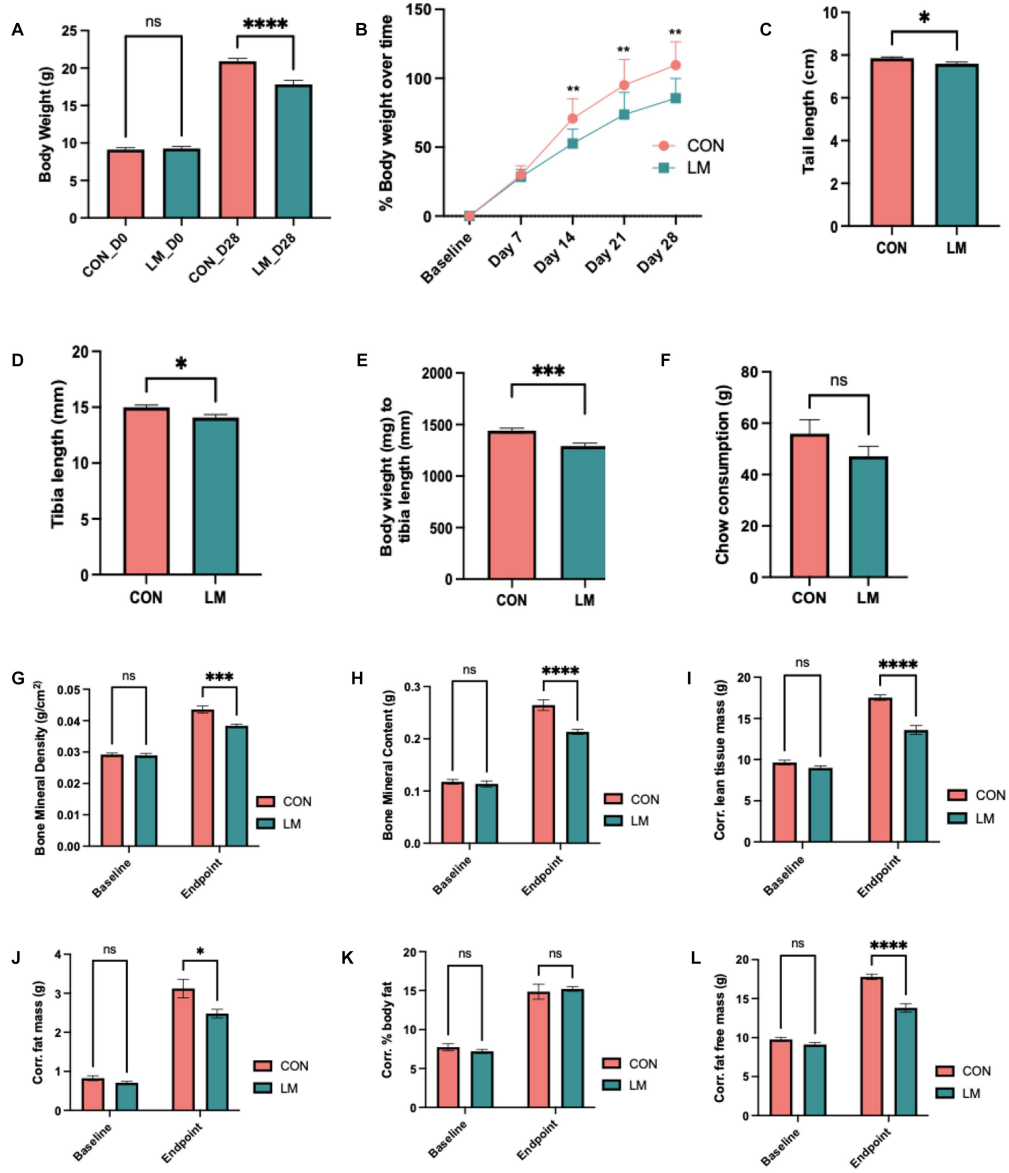
Impact of early life multiple micronutrient deficiencies on growth and body composition. **(A)** body weight, **(B)** percent weight gain over time, **(C)** average chow consumption **(D)** tail length **(E)**, tibia length **(F)** bodyweight/tibia ratio revealed stunting in the LM mice. Body composition assessed by DEXA scan Day 0 (i.e., baseline) and Day 28 showed lower **(G)** bone mineral density, **(H)** bone mineral content, **(I)** corrected lean tissue mass, **(J)** corrected fat free mass, **(K)** corrected percent body fat (ns), and **(L)** corrected fat mass at day 28. Data analyzed using Student’s *t*-test, and body composition using two-way ANOVA with Šidák multiple comparison testing. All data represented as mean ± SEM and *p* values expressed as *p < 0.05, **p < 0.01, ***p < 0.001, ****p < 0.0001, ns, not significant *p* > 0.05.

Next, we examined the impact of postnatal micronutrient deficiency on growth and body composition. Consistent with other malnutrition phenotype, postnatal mice exposed to multiple micronutrient deficiencies gained less bodyweight (*p* < 0.0001) and became stunted as characterized by shorter tail length (*p* = 0.02), and shortened tibia (*p* = 0.01) compared to controls, surrogate markers for stunting in mice (Figures 2A–D). Bodyweight to tibia length ratio was also lower in the LM group (*p* < 0.001) (Figure 2E), despite no significant difference in chow consumed (total consumed by group on Day 28) (Figure 2F).

DEXA scan showed altered body composition in the LM group. Both bone mineral density and bone mineral content (*p* < 0.0001, both) were significantly depleted in the LM group at Day 28. Although no difference was observed in percent body fat, other markers of future metabolic disease risk were significantly reduced in the LM group at Day 28 [fat free mass (FFM, *p* < 0.0001)], lean tissue mass (LTM, *p* < 0.0001), and fat mass [(FM, *p* < 0.01) (Figures 2G–L)]. In summary, our model showed substantially altered growth and body composition in response to the low-micronutrient diet.

### Multiple micronutrient deficiencies induce glucose dysregulation

**Figure 3:**
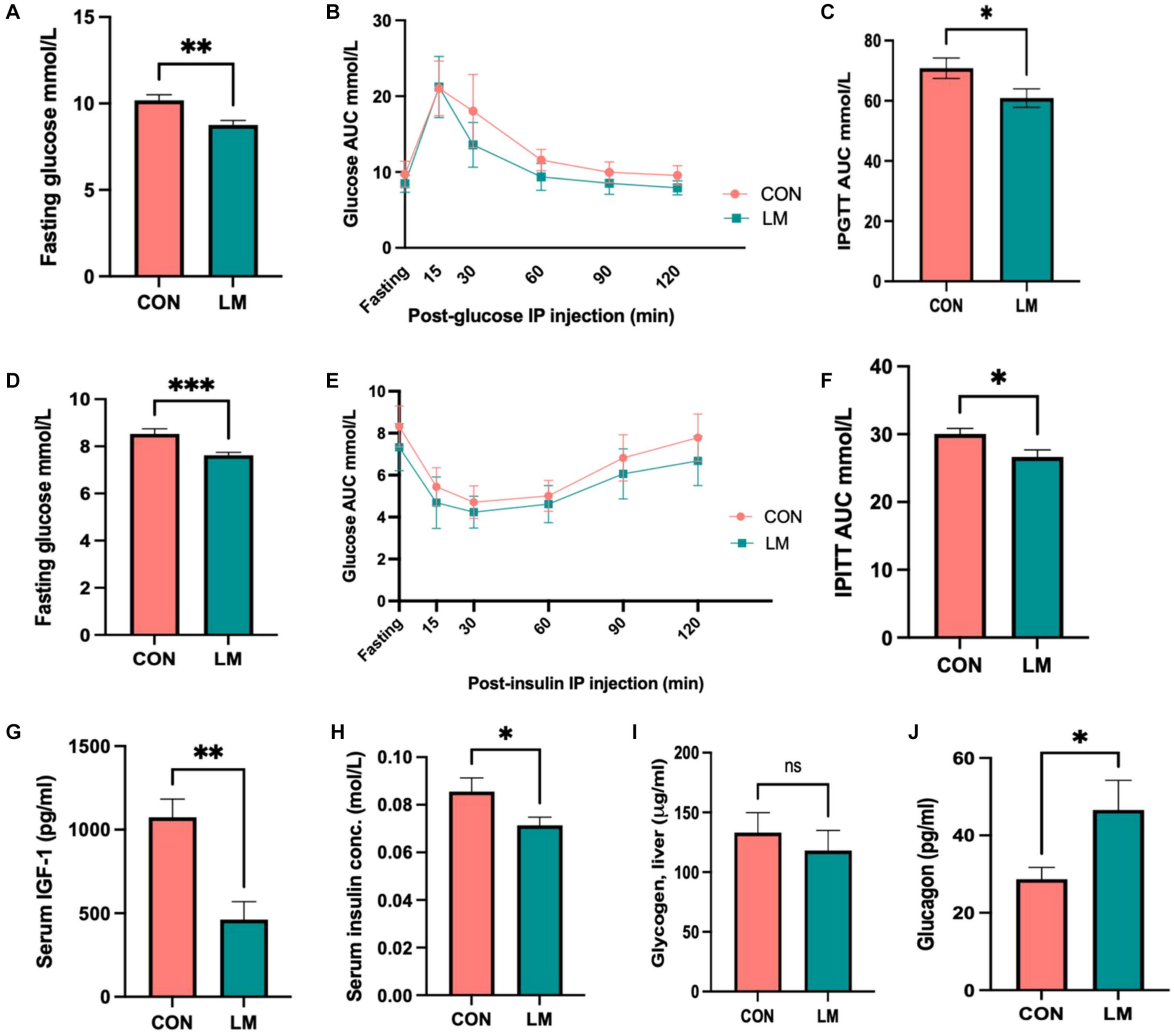
Glucose metabolism dysregulated in mice fed the low-micronutrient diet. **(A–F)** Fasting glucose, intraperitoneal glucose tolerance test (IPGTT) area under the curve (AUC), intraperitoneal insulin tolerance test (IPITT) AUC. **(G–J)** IGF-1, serum insulin, liver glycogen storage periodic acid-Schiff stain, (data not shown), glycogen assay and glucagon were assessed. Data shown here are from two independent experiments of 10 mice/group. Glucose and Insulin data are analyzed using ANOVA with *post hoc* Tukey’s test. All other data analyzed using Student’s *t*-test and values expressed in mean ± SEM. *p < 0.05, **p < 0.01, ***p < 0.001, ****p < 0.0001, ns, not significant *p* > 0.05.

Although aberrant glucose metabolism has been shown to be associated with some severe acute malnutrition survivors later in life, this link has not been explored in the case of multiple micronutrient deficiencies (19, 20, 29). Thus, we investigated the impact of early-life micronutrient deficiency on glucose and insulin metabolism. We consistently found lower (*p* < 0.0001) fasting glucose (FG) concentrations in the LM group (Figures 3A,D). Intraperitoneal glucose tolerance tests (IPGTT) demonstrated that the normal rise in blood glucose that should occur post-injection at the measured time points (15, 30, 60, 90, and 120 min) was significantly lower in the LM group. Area under the curve (AUC) quantification confirmed that these findings remained consistent across multiple independent experiments (*p* < 0.001) (Figures 3B,C). When challenged with an intraperitoneal insulin tolerance test (IPITT), mice fed the low micronutrient diet showed lower glucose levels at each time point and overall (*p* < 0.001; AUC) (Figures 3E,F). Since insulin and IGF-1 are major hormones that play roles in both linear growth and glycemic control (30), we next measured insulin and IGF-1 concentrations. Both showed lower levels in serum of the LM group (*p* < 0.05) (Figures 3G,H). However, serum pro-insulin and c-peptide levels, markers of insulin production, were not different between the groups (*P* = n/s). Further, no difference was found in serum insulin degrading enzyme, an enzyme involved in insulin clearance (Supplementary Figure S2).

We further examined several pathways regulating glucose homeostasis, namely glycogenesis, and gluconeogenesis. We did not find evidence of increased glycogen storage (Figure 3I) in the liver upon periodic acid-Schiff (PAS) stain (data not shown), and glycogen assay (*P* = n/s for both). Next, we examined glucagon, one of the key stimulators of hepatic glucose production (31). Our results showed higher fasting levels of glucagon (*p* < 0.05) in the LM group (Figure 3J).

**Figure 4:**
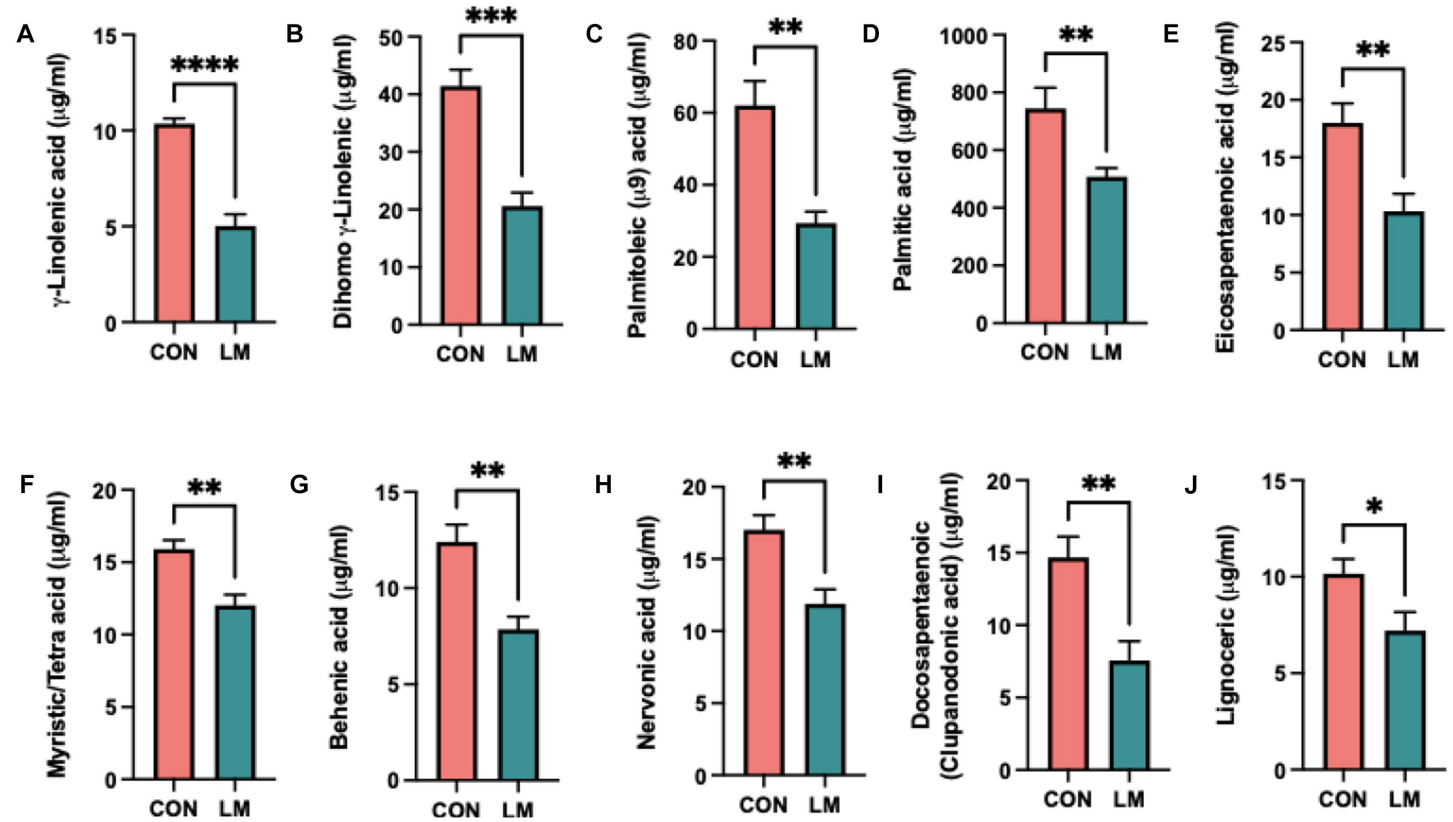
Postnatal multiple micronutrient deficiencies alter non-esterified free fatty acids (NEFA). **(A–J)** Significantly lower amount of free fatty acid found in serum of low micronutrient mice γ-linolenic, dihimo γ-linoleic acid, palmitoleic, palmitic, eicosapentaenoic, myristic/tetra, behenic, nervonic, and docosapentaenoic (clupanodonic) acids and lignoceric acid. Data represents 6 mice per group and the mean value represented as ± SEM. All data were analyzed using Student’s *t*-test and values represented as *p < 0.05, **p < 0.01, ***p < 0.001, ****p < 0.0001, ns, not significant *p* > 0.05.

Altered lipid phenotype in ten out of the thirty-one free fatty acids (FFA) measured was found. We saw markedly lower non-esterified free-fatty acids (NEFA), specifically γ-linolenic, dihomo-γ-linolenic, palmitoleic, palmitic, eicosapentaenoic, myristic/tetra, behemic, nervonic, docosapentaenoic and lignoceric acids in the serum of LM mice (Figures 4A–J). Gamma-linolenic acid was the least abundant FFA, amounting to 50% less than controls (5.4 μg/mL, 10.4 μg/mL, respectively). We did not find any difference in the omega-6 (ω-6) to omega-3 (ω-3) ratio (P = ns, calculation not shown). Among the significant FFAs, polyunsaturated fatty acids (PUFAs) tended to be lower than monosaturated fatty acids (MUFAs) and saturated fatty acids in both groups. The complete list of results is provided (Supplementary Table S1). Overall, we showed that energy metabolism was dysregulated in response to postnatal exposure to a multiple micronutrient-deficient diet.

### Early life micronutrient deficiency alters gut microbiome functional pathways and SCFA profile

**Figure 5:**
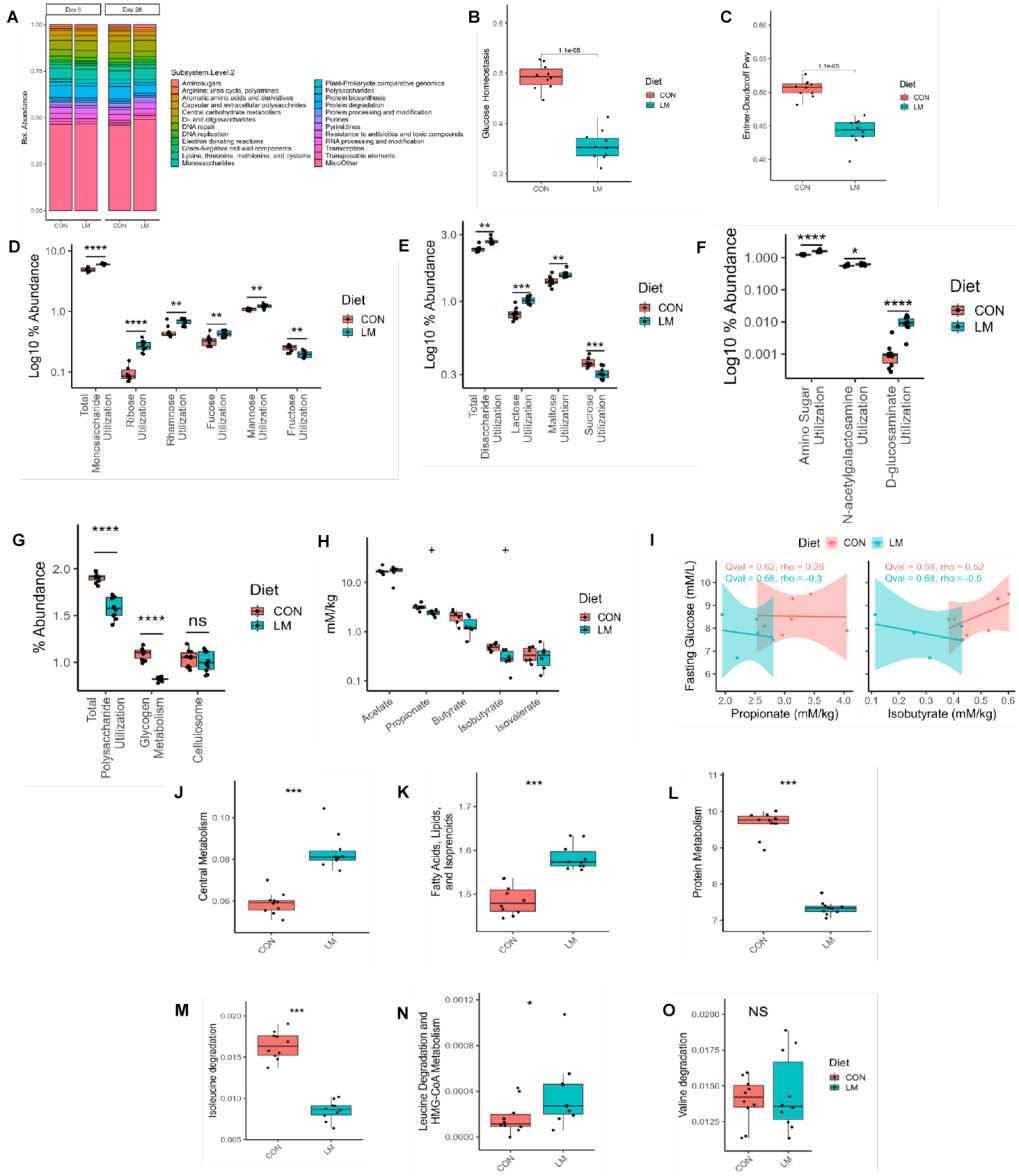
Early life micronutrient deficiency alters gut microbiome functional pathways and SCFA profile. **(A)** Functional pathway bar plot. **(B)** Glucose homeostasis, **(C)** Entner-Doudoroff, **(D)** monosaccharides utilization, **(E)** disaccharide utilization, **(F)** amino sugar utilization, **(G)** polysaccharide utilization, **(H)** Short-chain fatty acid analysis. **(I)** SCFA and fasting glucose correlation analysis. Gut microbiome functional pathway analysis, central metabolism **(J)**, fatty acids **(K)**, protein metabolism **(L)**, branched-chain amino acid metabolism, isoleucine **(M)**, leucine **(N)** and valine **(O)**. All *y* axis values represent their percent abundance in the metagenomic functional dataset. Wilcoxon tests were used to calculate all *p* values, which were then FDR corrected. SCFA analysis represented as *q* values. Statistical values represented as *p < 0.05, **p < 0.01, ***p < 0.001, ****p < 0.0001, +*p* > 0.05, n/s, not significant. SCFA, short-chain fatty acid.

A paucity of data exists on the metabolic function of the maturing microbiome, especially within the context of undernutrition. We performed shotgun metagenomics at the start (Day 0) and end (Day 28) of the experiment and investigated pathways related to glucose metabolism in the gut microbiome. Our results revealed a shift in the functional metabolism of the gut microbiome on Day 28 (Figure 5A). Of interest, we observed lower relative abundance of genes for glucose homeostasis and Entner-Doudoroff (alternative energy pathway used by Gram-negative bacteria) pathways (*p* < 0.001) in the LM group (Figures 5B–C) (32). In the LM group, we found increased abundance of genes involved in utilization of monosaccharides (*p* < 0.001). Further analysis into this pathway showed enrichment of genes required to utilize simple sugars such as ribose (*p* < 0.001), rhamnose (*p* < 0.01) fucose pathway (*p* < 0.05) and mannose (*p* < 0.01). However, there was a loss in gene abundance for fructose (*p* < 0.01) (Figure 5D). Similarly, among disaccharides we found increased gene abundance for lactose (*p* < 0.001) and maltose utilization, but significantly less utilization for sucrose (*p* < 0.01) (Figure 5E). Pathways involved in utilization of amino-sugars (*p* < 0.001), namely N-acetylgalactosamine (*p* < 0.01) and D-glucosaminate (*p* < 0.001) were also increased (Figure 5F). Conversely, we saw decrease utilization for more complex polysaccharides (*p* < 0.001), namely glycogen metabolism (*p* < 0.001), but no difference in cellulosome in the LM group (Figure 5G). No difference was found in these sugars prior to dietary treatment at the Day 0 timepoint (Supplementary Figure S3).

We found a significant difference in other functional metabolic pathways, including increased capacity in the central metabolism pathway (i.e., carbohydrates, amino acids and fatty acid degradation) (*p* < 0.001), in the LM group (Figure 5J). Unlike the finding in the host, we observed increased functional capacity in fatty acids, lipids, and isoprenoid pathways (*p* < 0.001, Figure 5K). Conversely, in LM group, there was a decrease in protein metabolism (*p* < 0.001), and in the branched-chain amino acids isoleucine (*p* < 0.001), leucine (*p* < 0.05) but no difference in valine (ns) (Figures 5L–O). We further examined whether the metagenomic findings could be observed *in vitro*. For this end, we performed a carbohydrate fermentation assay on Day 28 fecal samples and examined the saccharolytic ability of the fecal microbiome exposed to several types of sugars, namely xylose, glucose, ribose, mannose, trehalose, maltose, lactose, and confirmed utilization indicated by pH change (i.e., change in media color) and gas production *via* accumulation in a Durham tube. Both groups consumed the sugars, producing organic acids which caused a reduction in pH changing the media from red to yellow (data not shown). However, we observed differences in the gas-by product produced by the bacteria from the LM group consuming sugars compared to the controls. Among the monosaccharides, mannose, and glucose were more greatly fermented, while maltose and lactose were more abundantly fermented among the disaccharides (Table 2).

**Table 2:** Carbohydrate fermentation assay.

Carbohydrate fermentation gas production							
Group	Glucose	Xylose	Trehalose	Mannose	Maltose	Lactose	Ribose
CON	15 mm	28 mm	50 mm	32 mm	2 mm	9 mm	30 mm
LM	22 mm	32 mm	50 mm	50 mm	25 mm	19 mm	28 mm

SCFAs are byproducts of carbohydrate fermentation and involved in energy metabolism which we further examined within our model. We found a non-significant trend (*q* value = 0.06) toward lower levels of propionic acid and the branched short-chain fatty acid (BSCFA) isobutyric acid in the LM group compared to the controls at Day 28. No difference was seen in other SCFAs (Figure 5H). We saw no correlation between these two SCFA and fasting blood glucose in mice (Figure 5I). Taken together, we found that a low-micronutrient diet alters the energy metabolism and functional output of the developing microbiome and has a marginal effect on SCFA production.

### Single zinc deficiency had no effect on growth and glucose metabolism compared to multiple deficiencies

**Figure 6:**
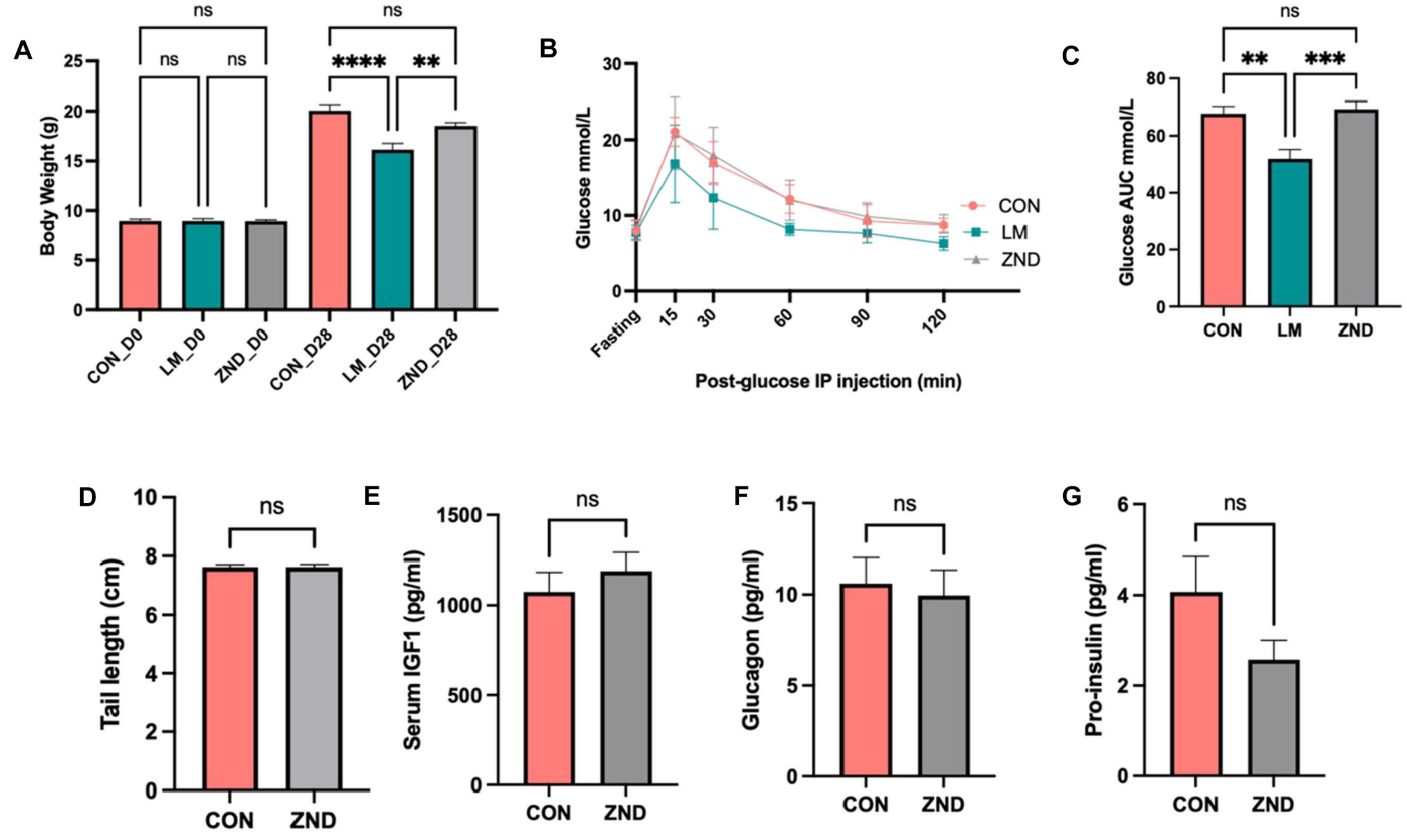
**(A)** Body weight comparison CON, LM, and ZND. **(B)** Intraperitoneal glucose tolerance test IPGTT, **(C)** IPGTT area under the curve bar plots. **(D)** Tail length CON and ZND, **(E)** Serum IGF1 CON and ZND, **(F)** Glucagon CON and ZND, **(G)** Pro-insulin CON and ZND. Data analyzed using ANOVA with *post hoc* Tukey’s test. All other data analyzed using Student’s t-test and values expressed in ±SEM. *p < 0.05, **p < 0.01, ***p < 0.001, ****p < 0.0001, n/s, not significant *p* > 0.05.

Zinc has been shown to play a role in stunting, glucose metabolism, and gut microbiota alteration (33, 34). Therefore we chose zinc as a likely candidate to examine the effect of a single micronutrient on these parameters. Zinc deficiency did not result in growth faltering or in glucose metabolism (Figures 6A–G).

## Discussion

Co-occurring micronutrient deficiencies are a global health problem that is vastly understudied. We developed a postnatal mouse model of multiple micronutrient deficiencies that addresses micronutrients of important public health concerns. Our results suggest that multiple micronutrient deficiencies result in physical and metabolic changes in the host and gut microbiome that is consistent with other malnutrition models and human cohorts (7, 23, 33, 35, 36). Surprisingly, we found simultaneous copper, selenium, and molybdenum deficiency and trapped manganese in the liver, yet all were not excluded from the diet. The findings of copper deficiency associated with zinc depletion are of particular interest as previous literature shows that the only known relationship between these two micronutrients is that high, and not low, zinc is associated with low copper by preventing its absorption (37). These intersecting relationships have not been previously described in an animal model or known in humans. Our findings underscore the critical need to study multiple micronutrients and the interaction between host and gut microbiome metabolic function as these nutrients act in concert. Moreover, the lack of difference in our zinc-only experiment on growth, glucose, and insulin metabolism and behavior further supports the need to study co-occurring rather than single deficiencies within the context of global undernutrition. A summary table of human studies and animal experiments with zinc deficiency and supplementation is provided in Supplementary Table S3.

Micronutrients are critically involved in energy metabolism directly or indirectly by acting as cofactors or coenzymes, specifically in glucose metabolism through endocrine, biochemical, and microbial pathways (34, 38). However, much remains to be understood about their causal relationship. Mice on the low-micronutrient diet consistently showed lower fasting glucose and lower circulating glucose levels within our model. While we hypothesized that impaired insulin clearance might play a role, we did not find evidence supporting this mechanism. An interesting follow-up would be to examine whether postnatal exposure to multiple micronutrient deficiencies increases the risk of metabolic disease later in life, where the current model could be adapted to emulate the double burden of malnutrition, which is described as the cooccurrence of multiple forms of malnutrition within the same individual (22). This model is currently under development.

Altered lipid metabolism, elevated or decreased levels, is frequently observed in malnourished children and animal models (23, 39, 40). Moreover, both dyslipidemia and higher lipid serum concentration in adulthood are correlated with early-life undernutrition in children (40). We did not alter lipids in our treatment group; thus, our findings suggest either malabsorption or dysfunction in the metabolism of lipids in mice fed a low-micronutrient diet. Our findings were consistent with lower PUFA profiles frequently found among children with severe acute malnutrition (SAM) and moderate acute malnutrition (MAM) (41). Interestingly, the PUFA profile in our model differed from SAM and MAM studies which are characteristic of lower arachidonic and docosahexaenoic acids as the main drivers in this model were γ-linoleic, dihimo γ-linoleic, eicosapentaenoic (EPA), and docosapentaenoic (clupanodonic) acids (DPA)This, however, may be due to several factors, the most predominant being the form of malnutrition. Severe acute and moderate acute malnutrition is caused by protein-energy deficiency, whereas our model focused on micronutrient deficiencies and was done in mice. Essential fatty acid deficiency (EFAD) is also common in children with severe acute malnutrition (41, 42). Given our fatty acid phenotype, our data suggest a potential onset of essential fatty acid disease within this mouse model, although this needs to be confirmed. Moreover, low plasma NEFA is indicative of suppressed lipolysis, which induces increased fat storage. Given that our DEXA did not show an increased fat mass in the low-micronutrient mice, the altered fatty acid may have also contributed indirectly to our aberrant glucose phenotype. Impaired fatty acid metabolism has also been shown to increase the risk of metabolic diseases, and our findings point to potential mechanisms of the developmental origins of disease in the undernourished (43). In a cohort of undernourished infants with environmental enteric dysfunction (EED) in rural Pakistan, Narvaez-Rivas and colleagues found altered NEFA metabolism and EFAD correlated with impaired growth in EED children. The infants also presented with EFAD linked to lower linoleic and n-6 PUFAs. Conversely, higher oleic acid was observed and suggested as a compensatory mechanism for dysregulated lipid metabolism (44). Additional follow-up studies examining NEFA over multiple time points and growth would shed additional insights. Furthermore, our model provides an excellent opportunity to elucidate the role of micronutrients in NEFA metabolism and EFAD in undernourished children. We conclude that different forms of malnutrition may select for different fatty acids, but the characteristic feature remains the same in the undernourished phenome, and our data complements findings in children.

Functional maturation of the microbiome is marked by its metabolic capacity, namely to utilize and degrade certain nutrients, like sugars or short-chain fatty acids, during different stages of development (21). Derrien et al. showed that the developing microbiome has a greater capacity for simple carbohydrate utilization, whereas functions to utilize and degrade complex carbohydrates (e.g., polysaccharides) are characteristic of a more mature community (21). Our data support an overall functional change in metabolism in the gut microbiome of the low-micronutrient-fed mice. Although we did not see any differences between the groups in our SCFA analysis, we may have been underpowered to detect these differences. SCFAs have been shown to play a central role in host energy metabolism. Propionate is the only SCFA substrate for gluconeogenesis in ruminants, where it is converted to glucose through the tricarboxylic acid (TCA) cycle, and studies have shown its role in glucose and insulin regulation (45, 46). We also found a strong trend toward lower isobutyric acid in the LM group. Isobutyrate is a branched short-chain fatty acid (BSCFA) produced through fermentation of branched-chain amino acids (BCAA), mainly by *Bacteroides* and *Clostridium* species (47). However, BSCFAs are less studied, and their function insufficiently understood. Nonetheless, BSCFA was recently investigated in energy metabolism and shown to modulate glucose and lipid metabolism in adipose tissue (48). SCFAs can act on hormones such as glucagon-like peptide-1 (GLP-1) and leptin to regulate host glucose (49). SCFAs are substrates and regulators of lipid metabolism. They, mainly butyrate and acetate, can activate fatty acid oxidation and inhibit lipolysis in adipose tissues, which impacts host energy (49, 50). Additional follow-up studies supplementing the diets with various SCFAs and examining adipose tissue and fatty acids via DEXA would be useful to examine their role in lipid and glucose metabolism in the micronutrient deficient host. Our data also showed that micronutrient deficiency significantly impacts reshaping metabolism within the gut microbiome. How this differs between species and which can gain fitness in such an environment awaits further exploration.

Early-life exposure to nutritional deficiencies has been linked to decreased neurocognitive function, including decreased verbal and motor skills, delayed learning, and spatial memory deficits in children (51). We investigated the impact of early-life multiple and single micronutrient malnutrition on neurocognitive outcomes in mice. Overall, we reported that mice in the LM group traveled less distance and alternated to different arms of the Y-maze than the CON and ZND mice; however, this was not significant after final adjustment calculations. It could be that the mice in the LM group had less exploration in general, which altered their final score. We also reported no difference in brain weights. Brain structure is formed *in-utero*, but the “wiring” (formation of neuronal connections) occurs throughout life; thus, differences may exist on a functional level not captured by the Y-maze.

### Limitations and future directions

Our model has the following limitations; first, while insightful, mouse models have limited translations to humans. Nonetheless, they provide valuable biological information that can be further assessed using humanized mouse models and *in vitro* and *ex vivo* experiments (e.g., human-derived organoids). We only used male mice in this current model, and although the original plan was to include females, the COVID-19 research disruption altered this course. Future work would benefit from examining sex differences as micronutrients are metabolized and utilized differently in females. We did, however, in our maternal model of micronutrient deficiency (Holani et al., manuscript under revision) which examined both sexes in pups and found no sex difference in growth. Other parameters remain to be explored. While our zinc-only deficient experiment showed no changes in host phenotype, our zinc level (2 mg/kg body weight) may not have been low enough to cause a change in growth and other functions. Future studies using 1 mg/kg may yield different results.

## Conclusion

Multiple micronutrient deficiencies remain a grossly understudied area of research. Global nutritional policies and interventions have been designed to address this condition, yet multiple micronutrient supplementation has only marginally delivered on its perceived promises (7, 8, 52, 53). In some cases, supplementation has improved micronutrient status but not growth. In other situations, high doses of multiple supplements had less impact than single supplements (8). While in others, deficiency persisted despite supplementation, suggesting our understanding of the underlying biology is incomplete. To our knowledge, animal models have not been used to guide many of these interventions or policies. As a result, the burden of malnutrition without the guidance of preclinical models persists, and the goal of achieving the World Food Program’s “Zero Hunger” initiative remains even further out of our reach. Salameh et al. have also proposed the use of undernutrition animal models as a useful tool for nutritional assessment and devising therapeutic strategies (54). Here, we both developed a model for postnatal multiple micronutrient deficiencies and simultaneously investigated mechanisms that may aid in our understanding of metabolic disease in host and microbiome. Our model provides an exciting opportunity to study cooccurring micronutrient deficiencies that complement clinical trials to guide interventions that target both the host and gut microbiome and exploration of mechanisms underscoring the Developmental Origins of Health and Disease within a multiple micronutrient deficient perspective.

## Data availability statement

The original contributions presented in the study are included in the article/Supplementary files, further inquiries can be directed to the corresponding author.

## Ethics statement

The animal study was reviewed and approved by University of British Columbia’s Animal Care Committee (ACC) and Canadian Council on Animal Care (CCAC).

## Author contributions

PL conceptualized and wrote manuscript, designed and executed experiment, and made figures. HB-Y edited manuscript and assisted with experiments. KE helped with experiments and edited manuscript. HL, CR-C, XH, PL, and KE performed all glucose measurements, analysis, and interpretation. RH assisted with experiments and editing manuscript. AM-R performed bioinformatics, figures, and edited manuscript. YF performed literature review. PL, YF, and TY performed the carbohydrate fermentation assay, assisted in writing the methods, imaging, and interpretation. NR performed the Y maze test, data analysis, and wrote methods. YF made zinc table. JJ helped draft the initial version and assisted with glucose metabolism interpretation. PL, HB-Y, and BF reviewed and approved final version of the manuscript. All authors contributed to the article and approved the submitted version.

## Funding

This work was supported by research grants from the Canadian Institutes of Health Research (CIHR) (BF) [FDN-159935] and grant from the Bill and Melinda Gates Foundation grant (OPP1170018). The funder has no involvement or restrictions regarding publication. BF is a University of British Columbia Peter Wall Distinguished Professor.

## Conflict of interest

The authors declare that the research was conducted in the absence of any commercial or financial relationships that could be construed as a potential conflict of interest.

## Publisher’s note

All claims expressed in this article are solely those of the authors and do not necessarily represent those of their affiliated organizations, or those of the publisher, the editors and the reviewers. Any product that may be evaluated in this article, or claim that may be made by its manufacturer, is not guaranteed or endorsed by the publisher.
