# Supplementary material for: Multiple micronutrient deficiencies alter energy metabolism in host and gut microbiome in an early-life murine model
Source: Front Nutr. 2023 Jul 10;10:1151670. doi: 10.3389/fnut.2023.1151670 (PMC10365968; doi:10.3389/fnut.2023.1151670)
Supplement: Supplementary file 1 [file Table_1.docx]

| **Ingredients** | **D21051302** | |
| --- | --- | --- |
|  | *Zinc Deficient Diet* | |
|  | gm% | *kcal%* |
| Protein (egg white) | 18.9 | *20* |
| Carbohydrate | 63.1 | *65* |
| Fat | 6.5 | *15* |
| Total |  | *100* |
| kcal/gm | 3.77 |  |
|  |  |  |
| **Ingredient** | **gm** | ***kcal*** |
| Casein | 0 | *0* |
| Egg White | 203 | *812* |
| L-Cystine | 0 | *0* |
|  |  |  |
| Corn Starch | 346 | *1384* |
| Maltodextrin 10 | 45 | *180* |
| Dextrose | 250 | *1000* |
| Sucrose | 0 | *0* |
|  |  |  |
| Cellulose, BW200 | 75 | *0* |
| Inulin | 25 | *25* |
| **Pectin, Tic Gums** | **0** | ***0*** |
|  |  |  |
| Soybean Oil | 70 | *630* |
|  |  |  |
| Mineral Mix S10026 | 0 | *0* |
| Mineral Mix S19427 (No Ca, P, K, Zn, or Fe) | 10 | *0* |
| Dicalcium Phosphate | 13 | *0* |
| Calcium Carbonate | 5.5 | *0* |
| Potassium Citrate, 1 H2O | 16.5 | *0* |
| Ferric Citrate (17.4% Fe) | 0.029 | *0* |
| Zinc Carbonate (52.1% Zn) | 0 | *0* |
|  |  |  |
| Vitamin Mix V10001 | 10 | *40* |
| Vitamin Mix V15927 (No vitamin A, Folate, or B12) | 0 | *0* |
| Vitamin Mix V15928 (350 IU A, 3 ug B12, 0.11 mg Folate) | 0 | *0* |
| Biotin, 1% | 0.4 | *0* |
|  |  |  |
| Choline Bitartrate | 2 | *0* |
|  |  |  |
| Pure Red Dye #40 | 0 | *0* |
| Pure Blue Dye #1 | 0 | *0* |
| Pure Yellow Dye #5 | 0.05 | *0* |
|  |  |  |
| **Total** | **1071.479** | ***4071*** |

**Supplemental Table 1: Dietary ingredients for zinc-only experiment.** Zinc only deficient diet was formulated by Research Diets Inc and was isocaloric with equal amounts of protein, carbohydrates and fats.
