# Supplementary material for: Multiple micronutrient deficiencies alter energy metabolism in host and gut microbiome in an early-life murine model
Source: Front Nutr. 2023 Jul 10;10:1151670. doi: 10.3389/fnut.2023.1151670 (PMC10365968; doi:10.3389/fnut.2023.1151670)
Supplement: Supplementary file 2 [file Table_2.docx]

|  | **Free fatty acid: microgram fatty acid per milliliter serum** | | | | | | | | | | | | | |
| --- | --- | --- | --- | --- | --- | --- | --- | --- | --- | --- | --- | --- | --- | --- |
| Lipids | Chemical name | C-1 | C-2 | C-3 | C-4 | C-5 | LM-1 | LM-2 | LM-3 | LM-4 | LM-5 | LM-6 | p-values | |
| Caproic acid | C 6:0 | 11.1 | 29.2 | 12.2 | 27.8 | 18.2 | 24.6 | 24.7 | 28.1 | 2.7 | 13.0 | 28.3 | 0.9282 | N/S |
| Caprylic acid | C 8:0 | 6.2 | 4.3 | 2.1 | 5.9 | 8.3 | 4.5 | 3.5 | 6.0 | 7.1 | 2.8 | 3.3 | 0.5105 | N/S |
| Capric acid | C 10:0 | 1.9 | 1.9 | 1.9 | 2.4 | 4.4 | 2.4 | 2.6 | 2.6 | 3.3 | 4.4 | 4.5 | 0.2223 | N/S |
| Lauric acid | C 12:0 | 16.7 | 18.8 | 7.3 | 22.6 | 38.4 | 24.5 | 20.9 | 32.7 | 33.3 | 24.6 | 30.1 | 0.2096 | N/S |
| Myristic/Tetra | C 14:0 | 15.3 | 15.6 | 16.0 | 14.4 | 18.2 | 9.7 | 12.4 | 10.7 | 15.1 | 12.3 | 11.9 | 0.0038 | ** |
| Myristoleic | C 14:1 | 4.7 | 3.3 | 1.3 | 0.9 | 0.9 | 5.2 | 2.4 | 2.2 | 1.3 | 1.4 | 1.4 | 0.9221 | N/S |
| Palmitic | C 16:0 | 571.4 | 741.9 | 662.7 | 1000.0 | 746.6 | 450.2 | 563.7 | 388.1 | 580.3 | 523.0 | 542.9 | 0.0099 | ** |
| palmitoleic | C 16:1n9 | 7.6 | 8.3 | 7.1 | 9.7 | 13.0 | 6.4 | 8.7 | 5.2 | 9.7 | 12.4 | 4.6 | 0.0012 | ** |
| palmitoleic | C 16:1n7 | 48.2 | 64.0 | 52.0 | 59.4 | 86.7 | 23.5 | 34.8 | 19.8 | 31.9 | 40.4 | 26.0 | 0.4493 | N/S |
| Stearic acid | C 18:0 | 267.6 | 311.8 | 291.7 | 327.8 | 350.0 | 235.9 | 293.7 | 202.0 | 346.3 | 282.3 | 261.5 | 0.1614 | N/S |
| oleic | C 18:1n9 | 314.9 | 452.0 | 330.1 | 426.6 | 509.5 | 246.4 | 331.5 | 201.9 | 348.3 | 381.9 | 336.8 | 0.058 | N/S |
| Vaccenic | C 18:1n7 | 48.1 | 60.6 | 44.3 | 54.0 | 79.1 | 28.9 | 39.8 | 24.4 | 48.1 | 54.0 | 46.6 | 0.0533 | N/S |
| Linoleic | C 18:2n6 | 726.2 | 861.5 | 843.0 | 938.6 | 956.7 | 669.5 | 684.3 | 548.6 | 951.3 | 649.5 | 877.7 | 0.1176 | N/S |
| Arachidic | C 20:0 | 2.8 | 8.8 | 7.1 | 16.8 | 7.9 | 3.0 | 9.8 | 2.0 | 6.8 | 5.7 | 3.1 | 0.1739 | N/S |
| γ-Linolenic | C 18:3n6 | 9.5 | 10.3 | 10.6 | 11.2 | 10.2 | 7.5 | 5.3 | 3.8 | 5.4 | 5.0 | 3.1 | <0.0001 | **** |
| Eicosenoic acid | C 20:1 | 6.5 | 0.0 | 0.0 | 0.0 | 0.0 | 0.0 | 0.0 | 0.0 | 5.8 | 0.0 | 0.0 | 0.8383 | N/S |
| Eicosenoic acid | C 20:1 | 0.0 | 2.6 | 8.8 | 11.1 | 11.2 | 5.0 | 8.7 | 6.2 | 5.2 | 7.9 | 7.8 | 0.9787 | N/S |
| α-Linolenic acid | C 18:3n3 | 20.6 | 23.7 | 28.0 | 29.0 | 35.1 | 24.0 | 29.2 | 18.4 | 40.9 | 21.5 | 35.5 | 0.8340 | N/S |
| Stearidonic acid | C 18:4n3 | 0.0 | 0.0 | 0.0 | 0.0 | 0.0 | 0.0 | 0.0 | 0.0 | 0.0 | 0.0 | 0.0 | No value |  |
| Eicosadienoic acid | C 20:2n6 | 4.8 | 7.3 | 5.0 | 7.2 | 7.3 | 3.7 | 5.8 | 3.6 | 6.3 | 6.2 | 8.6 | 0.5478 | N/S |
| mead acid (aka eicosatrienoic) | C 20:3n9 | 1.3 | 4.9 | 2.3 | 2.5 | 3.1 | 1.9 | 2.2 | 1.3 | 4.1 | 2.2 | 2.9 | 0.5901 | N/S |
| behenic acid | C 22:0 | 10.3 | 12.4 | 12.6 | 15.6 | 11.1 | 8.8 | 9.8 | 5.4 | 8.8 | 7.7 | 6.6 | 0.0025 | ** |
| Dihomo-γ-linolenic acid | C 20:3n6 | 35.0 | 50.1 | 35.8 | 41.3 | 45.0 | 16.3 | 18.5 | 13.7 | 27.5 | 27.4 | 20.1 | 0.0003 | *** |
| Arachidonic | C 20:4n6 | 319.7 | 368.2 | 278.8 | 460.9 | 447.8 | 243.4 | 356.5 | 183.5 | 346.9 | 288.1 | 272.8 | 0.06 | N/S |
| Eicosapentaenoic acid | C 20:5n3 | 16.2 | 18.6 | 14.6 | 16.3 | 24.3 | 9.1 | 13.1 | 6.8 | 14.9 | 12.5 | 5.5 | 0.0084 | ** |
| Lignoceric | C 24:0 | 7.8 | 10.1 | 9.5 | 11.1 | 12.3 | 5.5 | 9.5 | 4.0 | 10.2 | 7.6 | 6.4 | 0.0449 | * |
| Nervonic acid | C 24:1 | 13.5 | 18.2 | 16.0 | 18.1 | 19.3 | 10.0 | 11.7 | 8.5 | 14.5 | 14.8 | 11.8 | 0.0063 | ** |
| Adrenic acid  /Docosatetraenoic | C 22:4n6 | 5.5 | 6.8 | 4.7 | 5.7 | 8.2 | 5.3 | 6.8 | 3.5 | 6.3 | 5.2 | 7.7 | 0.6686 | N/S |
| Docosapentaenoic acid (Osbond acid) | C 22:5n6 | 8.8 | 14.9 | 7.7 | 7.2 | 9.9 | 5.7 | 9.1 | 2.8 | 7.5 | 7.7 | 14.9 | 0.4483 | N/S |
| Docosapentaenoic acid ( Clupanodonic acid) | C 22:5n3 | 12.3 | 16.6 | 10.7 | 15.2 | 18.6 | 7.7 | 10.5 | 7.4 | 1.7 | 10.8 | 7.2 | 0.0055 | ** |
| cis-4,7,10,13,16,19-docosahexaenoic acid | C 22:6n3 | 149.6 | 172.4 | 136.7 | 205.8 | 214.4 | 113.2 | 176.6 | 86.5 | 196.5 | 157.4 | 119.4 | 0.1781 | N/S |
| **Total** |  | 2664.2 | 3318.9 | 2860.6 | 3764.9 | 3716.1 | 2202.1 | 2706.0 | 1829.7 | 3088.0 | 2589.7 | 2709.2 |  |  |

**Supplemental Table 2: Free fatty acids metabolomics profile.** Non-esterified free fatty acids were measured in serum using mass spectrometry at the end of the experiment (i.e., Day 28). Results here show all fatty acids in micrograms per milliliter of serum.
