# Supplementary material for: Multiple micronutrient deficiencies alter energy metabolism in host and gut microbiome in an early-life murine model
Source: Front Nutr. 2023 Jul 10;10:1151670. doi: 10.3389/fnut.2023.1151670 (PMC10365968; doi:10.3389/fnut.2023.1151670)
Supplement: Supplementary file 3 [file Table_3.docx]

| Experimental summary | Type of diets (deficiency/supplement) | Effect on growth/stunting | Effect on glucose/insulin metabolism | Effect on gut microbiota | Reference |
| --- | --- | --- | --- | --- | --- |
| Mouse C2C12 cells and human skeletal muscle cells were were treated with insulin (10 nM), zinc (20 μM) and/or zinc pyrithione (10 μM) for 60 minutes. | Supplement | Not examined | Activation of insulin-dependent cell signaling molecules (Akt, Tyrosine, SHP, ERK1/2, PRAS40)  Glucose oxidation + | Not examined | Norouzi *et al.*(57) |
| Male 1-day old Cobb 500 broiler chicks were either unchallenged, challenged with *S. typhimurium* infection, or challenged with *S. typhimurium* infection and supplemented with 120 mg/kg of zinc for 42 days. | Supplement | Increased body weight gain; increased feed intake | Not examined | *Lactobacillus +*  *Clostridium populeti –*  Total bacteria + | Shao *et al.*(58) |
| Chicks upon hatching were fed with either Zn+ diet (42 μg/g zinc) or Zn- diet (2.5 μg/g zinc) for 4 weeks. | Deficiency | Not examined | Not examined | **Phylum level:**  *Proteobacteria +*  *Firmicutes –*  *Bacteroidetes +*  *Actinobacteria –*  **Family level:**  *Peptostreptococcaceae –*  *unclassified Clostridiales –*  *Enterococcaceae +*  *Enterobacteriaceae +*  **Genus level:**  *unclassified Peptostreptococcaceae–*  *Enterococcus +*  unclassified *Enterobacteriaceae +*  unclassified *Ruminococcaceae +*  (16S) | Reed *et al.*(59) |
| Male 22-day old C57BL/6 mice were fed on a protein source-defined normal diet and defined zinc deficient diet (<2 ppm zinc, 20% protein) for 14 days. | Deficiency | Similar growth with normal mice | Not examined | Low diversity measures | Mayneris-Perxachs *et al.* (37) |
| Female 8-week old C57BL/6JRj mice were fed zinc-adequate (41 mg/kg zinc), deficient in zinc (19 mg/kg zinc), or adequate in zinc but high in zinc uptake antagonists (41 mg/kg zinc + 41 mg/kg ZnAA supplements) diets for 8 weeks. | Deficiency | Not examined | Not examined | **Phylum level:**  *Proteobacteria –*  *Verrucomicrobia –*  *Firmicutes +*  *Bacteroidetes +*  **Family level:**  *Lachnospiraceae +*  **Genus level:**  *Bifidobacterium +*  *Eggerthella +*  *Odoribacter +*  *Eubacterium +*  *Anaerostipes +*  *Lachnoclostridium +*  *Murimonas +*  Appearance of *Turicibacter, Allobaculum, Marvinbryantia, and Butyrivibrio*  (16S) | Sauer *et al.*(60) |
| Male 25-d old Sprague-Dawley outbred rats were fed either adequate (55 mg/kg) or severely deficient (0.4 mg/kg) zinc diet for 25 days. | Deficiency | Low body and muscle weight | Not examined | Not examined | Giugliano *et al.* (61) |
| Rats were fed with Zinc deficient (< 1 p.p.m) or control diet (6 p.p.m). | Deficiency | Not examined | Blood glucose after glucose injection +  Insulin response  *–* | Not examined | Quarterman *et al.* (62) |
| 208 crossbred piglets were supplemented with 0 or 3100 mg/kg for 20 days post weaning. | Supplement | Not examined | Not examined | Small intestine tissue anaerobes + | Broom *et al.* (63) |
| 67 out of 177 school-age children were chosen to explore gut microbiome based on surveys and serum testing. | Deficiency | Not examined | Not examined | *Coprobacter* +  *Acetivibrio +*  *Paraprevotella* +  *Clostridium XI* + | Chen *et al.* (64) |
| 50 pre-pubertal Egyptian children with short stature and Zinc deficiency were given Zinc supplementation (50 mg/day) for 3 months. | Supplement | Serum IGF-1 +  IGF-1 SDS +  Serum IGFBP-3 + | Not examined | Not examined | Hamza *et al.* (65) |
| 30 pre-pubertal Brazilian eutrophic children were orally supplemented with 5 mg/day Zinc and injected with 0.06537 mg/kg body weight Zinc before and after oral supplements for 3 months. | Supplement | IGF-1 +  IGFBP-3 +  GH no significant difference | Not examined | Not examined | Alves *et al.* (66) |
| Healthy early adolescent girls were supplemented with 0 (control) or 9 mg/day Zinc for 4 weeks. | Supplement | Not examined | No significant effect in C-peptide, insulin, or HOMA2-IR levels | Not examined | Lobene *et al.* (67) |
| Three-week old male mice fed a zinc deficient diet 2mg/kg body weight for 28 days | Deficiency | No significant change on growth/stunting/body weight or IGF-1 between zinc deficient mice and controls | No significant effect on glucose or insulin | Not examined | Littlejohn et al. |

**Supplemental Table 3: Brief summary of zinc-deficient and zinc-supplemental studies in humans and animals.**
