# Supplementary figures and images for: Multiple micronutrient deficiencies alter energy metabolism in host and gut microbiome in an early-life murine model
Source: Front Nutr. 2023 Jul 10;10:1151670. doi: 10.3389/fnut.2023.1151670 (PMC10365968; doi:10.3389/fnut.2023.1151670)

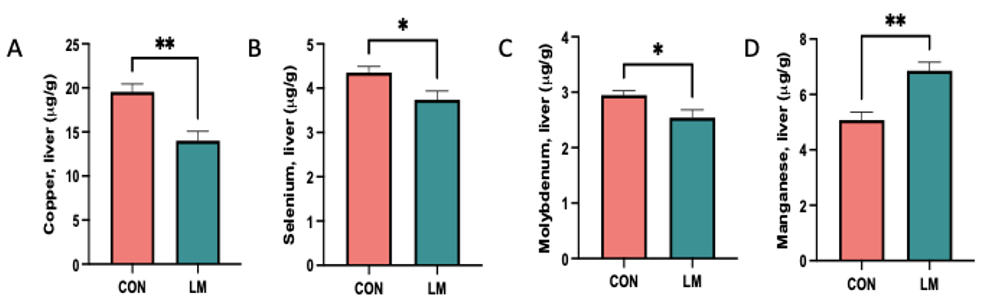

Supplement: Supplementary file 4 [file Image_1.JPEG]

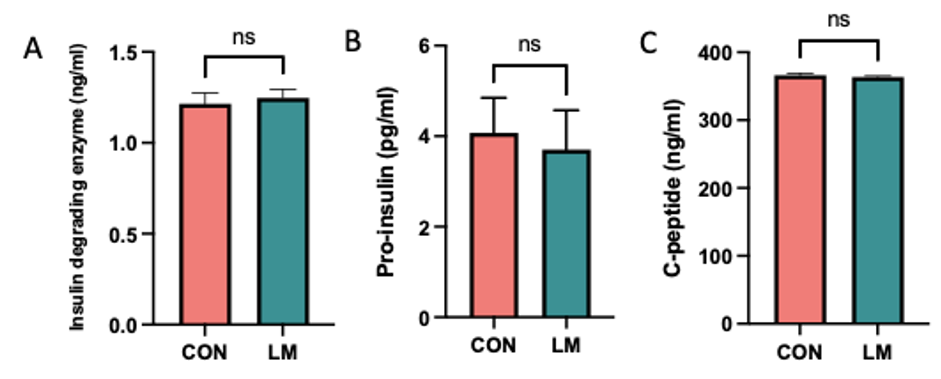

Supplement: Supplementary file 5 [file Image_2.JPEG]

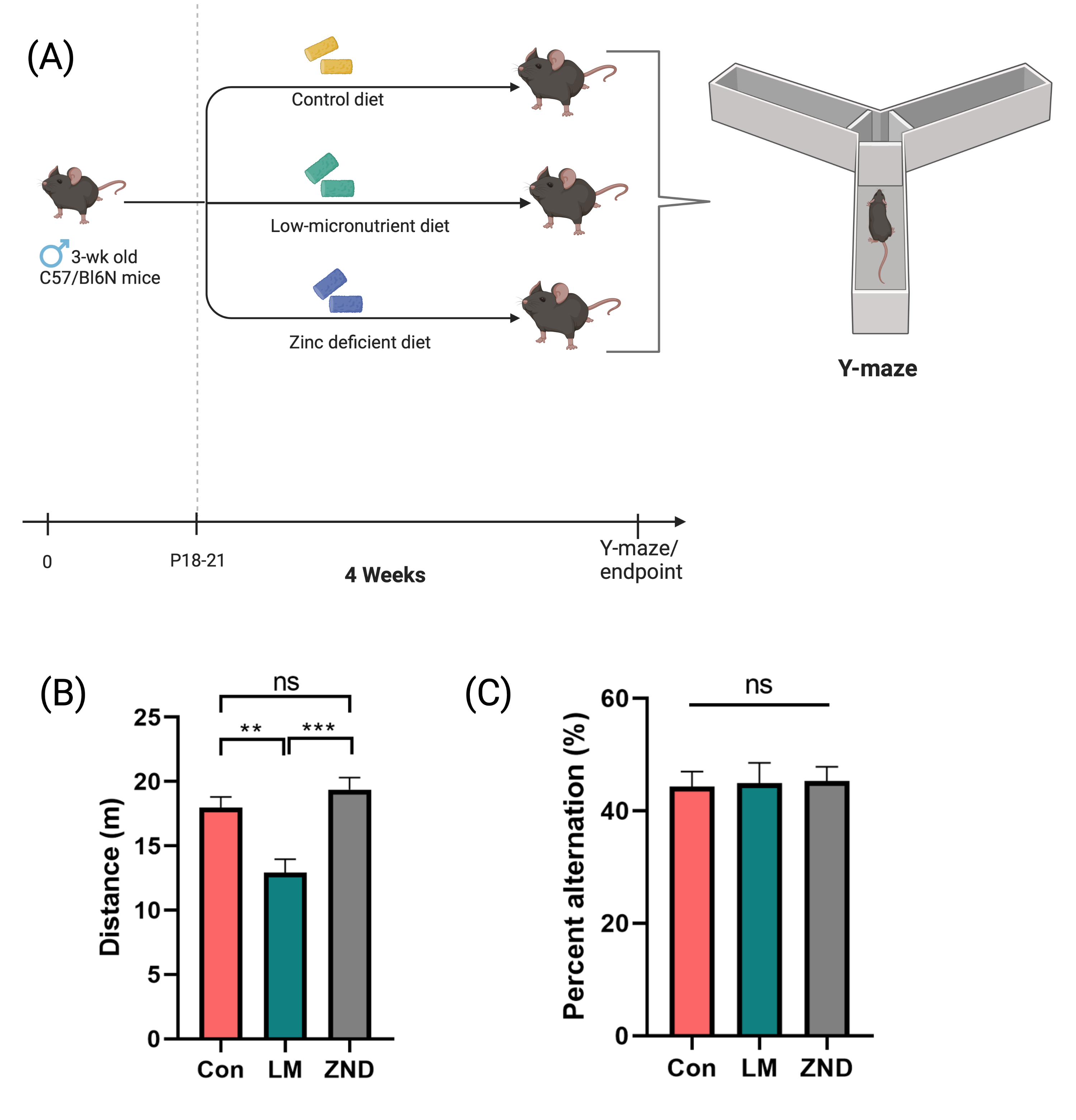

Supplement: Supplementary file 6 [file Image_3.jpg]
